# Structure-Based Comparative Metabolomics Identifies LysoPE 15:0 as a Candidate Metabolite Marker of Influenza Virus Infection Dynamics

**DOI:** 10.3390/molecules31132275

**Published:** 2026-06-29

**Authors:** Junxiao Wang, Yuting Li, Bin Wang, Wenxia Fang, Yushen Du, Fei Xu

**Affiliations:** 1 Zhejiang Provincial Key Laboratory of Synthetic Biotechnology for Microbial Medicine, Department of Gastroenterology, Second Affiliated Hospital, Zhejiang University, Hangzhou 310058, China; 15092094078@163.com; 2 Cancer Institute, Second Affiliated Hospital, School of Medicine, Zhejiang University, Hangzhou 310058, China; 12218164@zju.edu.cn; 3 Institute of Biological Science and Technology, Guangxi Academy of Sciences, Nanning 530007, China; bwang@gxas.cn (B.W.); wfang@gxas.cn (W.F.)

**Keywords:** structure-based comparative metabolomics, influenza virus, candidate metabolite marker discovery

## Abstract

Influenza virus outbreaks remain a persistent public health concern, yet traditional metabolomics methods are inadequate for addressing key analytical challenges of “dark matter” in influenza research. By integrating quantitative MS^1^ data, MS^2^-derived fragmentation trees and molecular fingerprints, structure-based comparative metabolomics enhances predictive capability for chemical structures, and enables the discovery of candidate metabolic markers without the need for database spectra. In this study, we established a C57BL/6J mouse model of H1N1 infection (with PBS as control) and performed structure-based comparative metabolomics on fecal samples using liquid chromatography–mass spectrometry (LC-MS). Quantitative analysis of MS^1^ data identified 40 differential metabolites, while qualitative analysis of MS^2^ data enabled their structural annotation. A candidate metabolite marker, LysoPE 15:0, along with other potential metabolic markers, was annotated and validated using Mirror plot, CFM-ID, and sim-Rank-Network. Our findings demonstrate that structure-based comparative metabolomics enables library spectra-free annotation of metabolomic “dark matter” and provides a methodological workflow for discovering candidate metabolite markers in other diseases.

## 1. Introduction

Influenza virus outbreaks are a continuous public health issue; seasonal global epidemics caused by both influenza A viruses (IAVs) and influenza B viruses (IBVs) cause 300,000 to 500,000 deaths each year [1]. Clinical diagnosis and laboratory testing methods focus on molecular biology testing, antigen testing, virus isolation and cultivation, serological testing, etc.; however, these methods cannot simultaneously meet the requirements of rapid, on-site and high-sensitivity detection [2]. Metabolic markers serve as real-time snapshots of physiological states, directly reflecting the ultimate functional output of an organism under the influence of genetic, environmental, pathological, and pharmacological factors [3]. Research on influenza virus metabolic markers translates the complex processes of life and disease into chemical language, and by annotating the “dark matter” of the metabolome, such research can predict disease trajectories and provide a foundation for diagnostic strategies in an accurate and fast way [4,5,6,7,8].

Liquid chromatography–mass spectrometry (LC-MS), characterized by high resolution and high sensitivity, has become an ideal tool for metabolomics research [9]. Specifically, MS^1^ enables the unbiased detection of all ionizable compounds within a sample, thereby offering a global view of the entire metabolome and serving as the basis for label-free quantification strategies. In parallel, MS^2^ selects specific precursor ions for fragmentation, effectively suppressing chemical noise and background interference, greatly facilitate the inference of molecular structures [10,11]. However, traditional metabolomics annotation is performed by combining MS^1^ precursor mass matching with MS^2^ spectral similarity, and thus annotations are intrinsically restricted to compounds for which a reference spectrum (usually based on commercially available chemicals) is present in the library, rendering traditional metabolomics annotation inadequate for tackling key analytical challenges of “dark matter”. In contrast, by leveraging quantitative MS^1^ data, constructing fragmentation trees from MS^2^ fragmentation patterns, and transforming these features into molecular fingerprints, structure-based metabolomics enables the annotation of metabolites even when their spectral information is unavailable in current databases, markedly enhancing the capability to predict chemical structures based on metabolomics data [12].

Comparative metabolomics can comprehensively capture the changes of small-molecule metabolites within an organism [13,14]; through comparative analysis, statistically significant differential metabolites can be identified, thereby enabling the discovery of metabolic markers. Prior influenza virus comparative metabolomics research has predominantly concentrated on samples such as serum, intestinal contents, and lung tissue, with findings emphasizing alterations in metabolic profiles, metabolic pathways, and the transcriptome [15,16]. Notably, metabolic marker identification and accurate metabolite annotation serve as the core bridge connecting raw mass spectrometry data to biological meaning, representing a critical step in moving from observing differences to understanding mechanisms and ultimately to clinical application, yet this priority has not been accorded the same level of attention mentioned above.

In light of the research background described above, this study employed a structure-based comparative metabolomics strategy. Using PBS/H1N1-infected mouse models and MS^1^/MS^2^ data interpretation via FUNEL [17], MetaboAnalyst [18], SIRIUS [19,20], and CANOPUS [9], we identified LysoPE 15:0 as a candidate metabolite marker of H1N1 infection. We further validated its structure and those of other potential markers (long-chain fatty acids and bile acids) using Mirror plot [21,22], CFM-ID [23,24], and sim-Rank-Network [17]. Finally, based on our annotations, we conducted a temporal metabolomic analysis of potential metabolic markers. Our experimental findings validate the efficacy of structure-based comparative metabolomics for the annotation of “dark matter”.

## 2. Results

### 2.1. Establishment and Evaluation of the Animal Model

**Figure 1:**
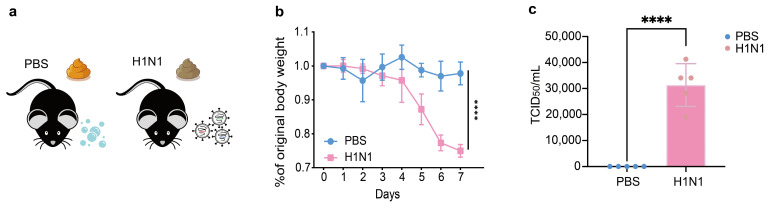
Establishment and evaluation of the animal model. (**a**) Groups of the mouse model used in this study. The control group (PBS) received phosphate-buffered saline, while the infection group (H1N1) was inoculated with the A/WSN/1933 influenza virus strain. (**b**) The weight-loss curves of mice in H1N1 and PBS groups from days 0–7. Mice in the H1N1 group (red line) exhibited progressive weight loss from day 2 to day 7 post infection, whereas the PBS control group (blue line) maintained stable body weight throughout the observation period. Data are presented as mean ± SD (*n* = 5). Statistical significance was determined by Student’s *t*-test. **** *p* < 0.0001. (**c**) Infectious viral titers in mouse lungs 7 days post infection. Lung samples were collected from PBS-treated and H1N1-infected mice 7 days post infection, and infectious viral titers were determined by TCID_50_ assay. Each dot represents an individual mouse (*n* = 5 per group), and bars indicate mean ± SD. Statistical significance was determined by Student’s *t*-test. **** *p* < 0.0001.

A growing body of evidence suggests that the lung-to-gut axis plays a critical role in respiratory tract infections, including influenza A virus infections [25,26,27]. Feces, compared with cecal contents, have the advantages of being relatively stable, easier to collect, more concentrated, and reflecting the overall output of the entire colonic bacteria, making them more suitable for metabolomics research on gut microbiota [28,29]. C57BL/6J female mice were randomly divided into a PBS-infected (PBS) group and an influenza A/WSN/1933-infected (H1N1) group, and fecal samples were subjected to LC-MS to collect MS^1^ and MS^2^ data for structure-based comparative metabolomics research (Figure 1a).

To evaluate the disease progression, we first monitored the body weight changes of infected animals. As shown in Figure 1b, the weight-loss curves of mice in the H1N1 group exhibited a continuous decline from day 0 to day 7. In contrast, the mock-infected group maintained a stable weight throughout the experiment. Next, pulmonary viral titers were determined by TCID_50_ assay. No infectious virus was detected in the PBS group, whereas H1N1-infected mice exhibited measurable pulmonary viral titers (Figure 1c). These data collectively confirm the successful establishment of the infection model.

### 2.2. Quantitative Analysis of MS^1^ Data Revealed 40 Differential Metabolites

**Figure 2:**
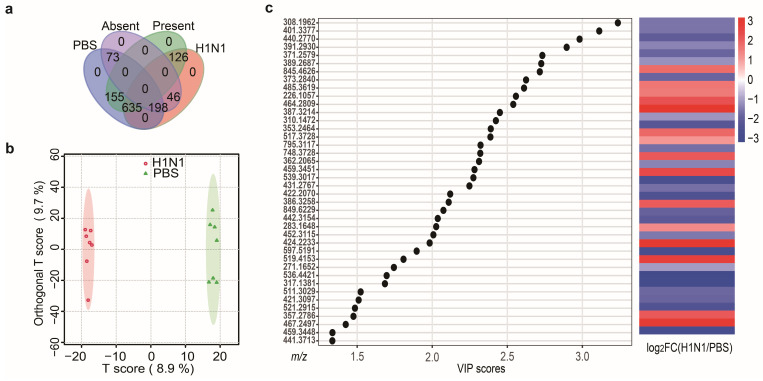
HMDB-search and quantitative analysis results of MS^1^ data. (**a**) Venn diagram showing the results of HMDB-search results for PBS and H1N1 groups. (**b**) OPLS-DA score plot. OPLS-DA score plot showing the metabolic trajectory separation between the PBS control group (green, right) and the H1N1-infected group (red, left) across days 1–7 post infection. Each data point represents an individual biological sample. The shaded ellipses indicate the 95% confidence intervals for each group. Two distinct clusters are clearly separated on opposite sides of the origin (0, 0). (**c**) VIP score plot and fold change heatmap of 40 putative metabolites with selection criteria of VIP >1, |log_2_FC| > 1, and unadjusted *p* < 0.05. The heatmap (right) visualizes the abundance changes of these metabolites between the H1N1-infected group and the PBS control group, where red represents upregulation and blue represents downregulation.

The HMDB (Human Metabolome Database) is currently the world’s most comprehensive and well-curated repository of human metabolite and metabolomics data. It serves as an indispensable core resource in metabolomics research, particularly for metabolite marker discovery and metabolite identification [30,31]. FUNEL [17] detected MS^1^ features for HMDB-searching, removed irrelevant chemicals and performed quantification of metabolites in the metabolome; as a result, 3602 *m*/*z* signals remained (Table 1). After removing signals with >50% missing values (corresponding to fewer than four occurrences within 7 days), 1233 putative metabolite signals were obtained, of which 317 *m*/*z* features lacked correspondence in the HMDB database, while the remaining 916 were matched to HMDB entries (Figure 2a), indicating that “dark matter” [4,5] still persists in H1N1 infection studies in metabolomics.

Later statistical analysis and differential metabolite screening were carried out by MetaboAnalyst [18]. The quality control (QC) samples clustered together in the principal component analysis (PCA) score plot (Figure S1), indicating good instrument stability and method reproducibility throughout the entire analytical run. The OPLS-DA score plot revealed a clear and complete separation between the PBS group and the H1N1 group (sampled from days 1 to 7), with the two distinct clusters located on opposite sides of the origin (0 point) (Figure 2b). This distinct separation demonstrates that the disease state induced a profound and consistent metabolic alteration, enabling the model to effectively differentiate between the two groups with high fidelity.

To identify metabolites potentially associated with H1N1 infection, we performed differential metabolite screening based on variable importance in projection (VIP) > 1 and absolute log_2_ fold change (|log_2_FC|) > 1. This initial filtering yielded more than 500 metabolites. Given our relatively limited sample size (*n* = 5 per group) and to avoid overly conservative adjustment for multiple comparisons, we did not apply strict multiple testing correction at this exploratory stage. Instead, candidate metabolites were ranked by their *p*-values (unadjusted *p*-values) to prioritize those with the stronger evidence of differential abundance. This approach allowed us to focus on the most promising metabolite candidates for subsequent temporal trajectory analysis. Under these criteria, a total of 40 metabolites showed significant alterations between the disease group and the healthy control group. Among these, 15 metabolites were significantly upregulated (log_2_FC > 1) and 25 metabolites were significantly downregulated (log_2_FC < −1) in the disease group compared to controls (Figure 2c). A full list of the identified differential metabolites, including their VIP scores, log_2_FC, and *p*-values, is provided in Table S1. Consequently, the follow-up research will center on these 40 differential metabolites.

### 2.3. Qualitative Analysis of MS^2^ Data Annotated the Structures of Significantly Differential Metabolites

#### 2.3.1. Structural Classification

**Figure 3:**
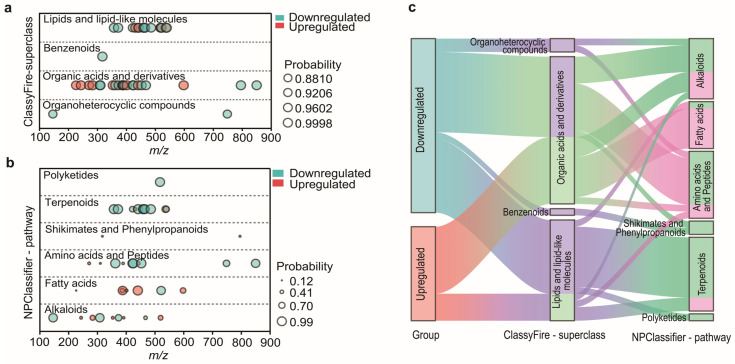
Structural classification results of differential metabolites. CANOPUS is built into the SIRIUS 5.8.6 software, and for each metabolite, it outputs the classification result with the highest score, detailed scores, etc. (**a**) ClassyFire’s results for superclass. (**b**) NPClassifier’s results for pathway. Red bubbles represent upregulated metabolites, while blue bubbles represent downregulated metabolites. Bubble size is probability. For each metabolite, CANOPUS generates a probability score ranging from 0 to 1, which represents the model’s confidence in its prediction that the metabolite belongs to a specific compound class. To ensure high-confidence annotations, only predictions with a probability > 0.5 were retained for downstream interpretation. Predictions with probabilities between 0.2 and 0.5 were considered low-to-moderate-confidence and were treated as tentative. (**c**) Sankey diagram of classification results. Nodes represent categories (compound superclass and pathway); links/edges represent flows or transitions between categories; and width of each link is number of metabolites.

Structural classification of compounds enables the simplification of complex metabolomics data interpretation, enables association with metabolic pathways, and improves the biological relevance of identified metabolite markers [32]. We performed structural classification of the 40 differential metabolites using CANOPUS [9], based on two independent classification schemes: ClassyFire [33] (a structure-based chemical taxonomy) and NPClassifier (a biosynthetically informed tool) [34]. Consequently, classification results were returned for 38 metabolites, while no results were obtained for precursors at *m*/*z* 511.3029 and *m*/*z* 845.4626, owing to the low-quality MS^2^ spectra. According to ClassyFire’s results, the differential metabolites were predominantly assigned to two superclasses—lipids and lipid-like molecules, and organic acids and derivatives—with high probability between 0.88 and 1.0 (Figure 3a, Table S2). NPClassifier classified the metabolites with high probability (score > 0.8) clearly revealing that fatty acids constituted the major class among upregulated metabolites, while terpenoids dominated the downregulated group (Figure 3b, Table S3). The Sankey diagram of dual validation improves reliability for the two classification schemes (Figure 3c), and enables interpretation of the 38 metabolites from both structural and biosynthetic perspectives. Our findings are in agreement with a previous overview, “The Role of Lipid Metabolism in Influenza A Virus Infection” [35].

#### 2.3.2. Structural Annotation

**Figure 4:**
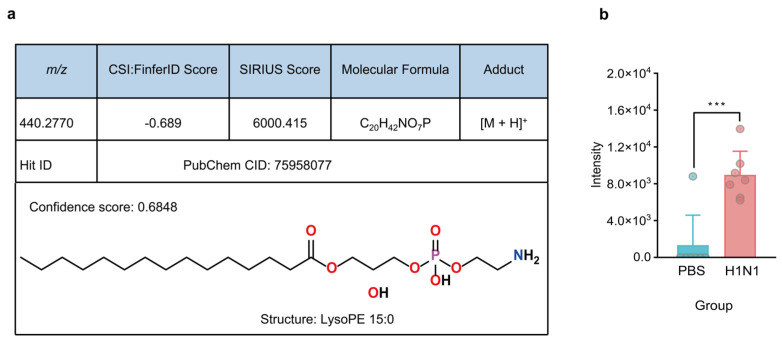
Detailed information of *m*/*z* 440.2770. (**a**) Structural annotation information of *m*/*z* 440.2770 from SIRIUS. (**b**) Abundance difference of *m*/*z* 440.2770 between PBS and H1N1 groups over days 1–7. Data are presented as mean ± SD (*n* = 7). The H1N1 group (red column) exhibited significantly higher abundance of *m*/*z* 440.2770 compared with the PBS control group (blue column) during days 1–7 (*** *p* < 0.001, Student’s *t*-test).

PubChem, the world’s largest chemical database comprising more than 119 million compounds, substantially enhances the probability of identifying potential candidate structures while preventing omissions that may arise from insufficient database coverage [36]. SIRIUS was used to construct fragmentation trees from MS^2^ fragmentation patterns, transforming these features into molecular fingerprints, and library retrieval in PubChem [19]. The SIRIUS structural annotation results revealed a distinct trend—upregulated metabolites were structurally enriched in long-chain saturated fatty acids, and downregulated metabolites in bile acids—which is consistent with the findings from the structural classification (Figures S2 and S3, Tables S2–S4). Furthermore, among the 18 differential metabolites annotated as lipids (bile acids and fatty acids), *m*/*z* 440.2770, annotated as LysoPE 15:0, achieved both a high confidence score (0.6848) and SIRIUS score (>6000) and a high CSI:FingerID score (−0.689) [37] (Figure 4a), and its abundance differed significantly between the PBS- and H1N1-infected groups (Figure 4b). LysoPE 15:0 is an endogenous compound arising from the host’s own metabolic pathways [19], and to date, LysoPE 15:0 has not been explicitly reported as a specific metabolite marker, whereas LysoPC 15:0 (sharing the same carbon chain length) and the entire LysoPE family have been documented to be significantly associated with disease states, including metabolic syndrome [38,39]. Consequently, LysoPE 15:0 emerges as a candidate disease metabolite marker.

### 2.4. Structural Verification of Candidate Metabolic Markers

**Figure 5:**
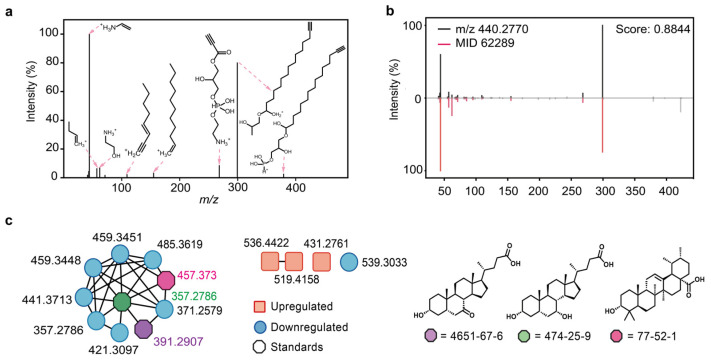
Structural verification of *m*/*z* 440.2770. (**a**) Peak assignment results from CFM-ID of *m*/*z* 440.2770; assignments for each major fragment ion are shown in the spectrum. (**b**) Mirror plot results for experimental spectrum versus reference spectrum of *m*/*z* 440.2770 with high cosine score. (**c**) sim-Rank-Network results of all predicted bile acids and chemical standards.

Unambiguous structural annotation is a prerequisite for targeted validation, which in turn enables the metabolite to be used as a clinical diagnostic metabolite marker. Due to the unavailability of a commercial standard, we performed peak assignment for *m*/*z* 440.2770 using CFM-ID [22,23], and all major MS^2^ fragment ions were rationally assigned (Figure 5a and Figures S4–S6, Table S5). Furthermore, we employed Mirror plot to compare the experimental spectrum with the reference spectrum of LysoPE 15:0 (MID 62289, an authentic standard spectrum) from a commercial database—METLIN [40]. Despite being collected on different mass spectrometry platforms with distinct parameters, the two MS^2^ spectra exhibited a high cosine similarity of 0.8844, with good alignment for fragment ions including 299.2566, 44.0492, etc. (Figure 5b and Figure S7). Cosine similarity scores higher than 0.7 are academically considered to indicate a high degree of spectral similarity, while scores greater than 0.8 can be regarded as reliable evidence for the confident identification of the same compound [41]. Our results match the feature *m*/*z* 440.2770 to LysoPE 15:0 with high confidence.

Although long-chain saturated fatty acids undergo insufficient fragmentation under collision-induced dissociation (CID) mode, bile acids generate informative MS^2^ fragment ions [42]. Therefore, three authentic bile acid standards were purchased (CAS 4651-67-6, 474-25-9 and 77-52-1), and a molecular network was constructed using simRank-Network [17] together with 11 differential metabolites annotated as bile acids by SIRIUS (Figure 5c). As results, 7 of 11 metabolites predicted to be bile acids were successfully clustered with the three purchased standards, and all clustered differential metabolites were downregulated in the disease group. This observation is in good agreement with previous findings that bile acid dysregulation plays a key role in H1N1 infection-associated pneumonitis and enteritis [8,43,44,45].

The failure to cluster the remaining four metabolites (*m*/*z* 539.3033, *m*/*z* 536.4222, *m*/*z* 519.4158 and *m*/*z* 439.2761, Figures S2 and S3) may be attributed to the following factors. First, the core structural backbone of the measured metabolites may differ considerably from that of the three standards, leading to low spectral similarity. Second, the fragment information generated under CID conditions may be insufficient to support reliable matching. Third, there are inherent limitations in the precision of the computational algorithm.

### 2.5. Temporal Metabolomic Analysis of Eight Metabolites

**Figure 6:**
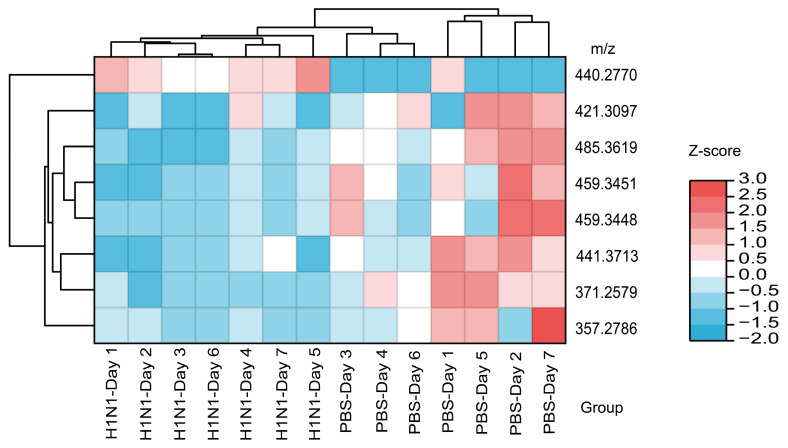
Temporal clustering heatmap of eight metabolites following H1N1 infection. Z-score normalized abundance is color-coded from blue (low) to red (high). Rows represent metabolites (*m*/*z*) and columns represent time points (days 1–7) for H1N1-infected and PBS-treated groups.

Temporal metabolomic analysis of potential metabolic markers allows for the direct visualization of the expression levels and dynamic trajectories of metabolites within each cluster [46]. Based on metabolite annotation results in our study, we conducted temporal metabolomic analysis of eight metabolites (one long-chain fatty acid *m*/*z* 440.2770 and seven other metabolites that successfully clustered with three bile acid standards). (Figure 6) Cluster heatmap analysis revealed that metabolite *m*/*z* 440.2770, which exhibited sustained upregulation in the H1N1 group but consistent downregulation in the PBS control group, represents a potential infection-specific candidate marker. Furthermore, H1N1 infection profoundly reshaped the temporal metabolic trajectories; metabolites that naturally increased over time in the control group, such as *m*/*z* 485.3619, were either suppressed or exhibited delayed elevation in the infected group. Notably, the unusually high levels of metabolite *m*/*z* 421.3097 observed on days 5 and 6 in the control group suggest that its variation may be influenced by non-infection-related factors.

## 3. Discussion

Quantifiable and dynamic in nature, metabolic biomarkers are small-molecule metabolites that act like chemical probes to read out the health or disease state of an organism. Consequently, they serve as a bridge connecting foundational studies of metabolism with real-world clinical applications. The advent of structure-based comparative metabolomics has markedly extended the boundaries of structural prediction for metabolomic “dark matter” [4,5]. Unlike traditional methods that rely on spectral library matching, this approach enables the annotation of metabolites even in the absence of reference spectra, thereby unlocking previously inaccessible regions of the chemical space.

Our research on influenza virus metabolic markers translates the complex processes of life and disease into chemical language, and a candidate disease metabolite marker LysoPE 15:0 [20] together with other two classes potential makers (long-chain fatty acids and bile acids) was identified during the progression of H1N1 infection. It should be emphasized that (i) to the best of our knowledge, the authentic measured LC-MS/MS spectrum for LysoPE 15:0 is not currently available in major databases, including PubChem [36] and HMDB [30,31] used in our research, and (ii) despite the limited sample size, *m*/*z* 440.2770 remained statistically significant after Benjamini–Hochberg (BH) correction, with a q-value of 0.02 (Table S1). The annotation of the feature at *m*/*z* 440.2770, therefore, provides compelling evidence for the superior performance of structure-based comparative metabolomics annotation.

Several limitations of this study should be acknowledged.

First, the sample size per group is relatively modest for untargeted metabolomics, which typically involves substantial biological variability among individuals. Moreover, pooling fecal samples from each day before LC-MS analysis makes it impossible to assess inter-individual variability or to apply mixed-effects models for correcting intra-animal correlation. To partially mitigate these limitations, we performed MS^1^-level putative annotation of the prioritized metabolites using the HMDB, matching *m*/*z* values within a mass tolerance of 0.01 Da. We further implemented stringent filtering criteria (detection rate > 50%, VIP > 1, |log_2_FC| > 1) and prioritized metabolites based on *p*-values. Nevertheless, these strategies cannot fully compensate for the lack of adequate sample sizes. Future studies with individual-level sampling will be necessary to confirm the robustness of these findings. Therefore, the candidate metabolites identified herein should be interpreted as exploratory, and their reliability warrants validation in subsequent targeted experiments as well as in independent study cohorts.

Second, LysoPE 15:0 is a lysophosphatidylethanolamine species that may reflect altered phospholipid metabolism during H1N1 infection. Lysophospholipids are known to modulate membrane fluidity, signaling pathways, and inflammatory responses. The significant elevation of LysoPE 15:0 in infected mice suggests its potential as a metabolic biomarker for influenza pathogenesis. According to the Metabolomics Standards Initiative (MSI) framework, the identification of LysoPE 15:0 is currently at Level 2 (putative annotation). Confirmation with an authentic standard under identical LC-MS conditions is required to upgrade the confidence to MSI Level 1. Given the exploratory nature of this untargeted metabolomics study and the modest sample size, further targeted validation and functional studies are warranted to confirm its biomarker utility.

Third, both tools are susceptible to false-positive annotations. SIRIUS may assign isomeric or structurally similar compounds when the correct structure is absent from training data—particularly for fatty acyls and similar compound classes. CANOPUS, while providing comprehensive compound class prediction across 1270 ChemOnt classes, has been trained on known structure databases; its accuracy may decrease for entirely novel or underrepresented chemotypes. Furthermore, CANOPUS predictions remain at the class level (e.g., “lipids” or “organic acids”) and do not substitute for definitive structural identification. Thus, advances in spectral libraries, machine learning-based prediction tools, and tandem mass spectrometry fragmentation techniques will be required to further close this gap.

## 4. Materials and Methods

### 4.1. Animal Model

Animal studies followed protocols approved by the Zhejiang University Institutional Animal Care and Use Committee (approval number: AIRB-2021-019). Ten C57BL/6J female mice (6–8 weeks of age) were purchased from Shanghai SLAC Laboratory Animal Co., Ltd. (Shanghai, China) and adapted to specific pathogen-free conditions for one week. Mice were randomly divided into two groups: a PBS-infected (PBS) group (*n* = 5) and an influenza A/WSN/1933-infected (H1N1) group (*n* = 5). Mice in the H1N1 group were intranasally inoculated once with 5 × 10^3^ TCID_50_ of influenza A/WSN/1933 virus in 50 μL sterile PBS per mouse, whereas control mice received 50 μL sterile PBS alone. Each mouse underwent one intranasal inoculation, and all mice in both groups were infected on the same day. All mice had access to water and food under a strict 12 h light/dark cycle.

The sample size of five mice per group was determined based on several considerations. First, this study was designed as an exploratory pilot investigation aimed at identifying potential metabolite signatures, rather than a definitive confirmatory study. Second, the sample size adheres to the 3R principle (Replacement, Reduction, Refinement) for animal research, minimizing animal usage while still enabling meaningful exploratory analysis. Third, this sample size was designed referring to previous studies of H1N1 infection in mice [47]. To address the analytical constraints imposed by the modest sample size (*n* = 5 per group), we implemented a multi-step strategy. First, MS^1^-level putative annotation of the prioritized metabolites was conducted using the HMDB by matching *m*/*z* values within a mass tolerance of 0.01 Da. Second, only metabolites detected in more than 50% of all samples were retained, ensuring analytical stability and reducing overfitting risk. Finally, candidate metabolites were prioritized using a combined threshold of variable importance in projection (VIP) > 1 and |log_2_FC| > 1, followed by ranking based on unadjusted *p*-values.

### 4.2. Sample Collection and Preparation

Body weight was measured and recorded for each animal on a daily basis, concurrent with the collection of fecal samples. These longitudinal weight data provided an essential reference for assessing the physiological condition of animals assigned to the healthy and disease groups.

Fresh fecal samples were collected at same time point each day for 7 days and stored at −80 °C. After thawing on ice, fecal samples from the same treatment group were pooled daily by taking 20 mg from each mouse, yielding seven pooled samples per group (one for each day of the 7-day period), and then 100 mg (±1%) of feces was added to 600 μL of methanol and then vortexed for 30 s at −20 °C. Next, the fecal samples were subjected to ultrasound at room temperature for 10 min after grinding with 100 mg glass beads for 90 s at 60 Hz. Then, the samples were centrifuged at 12,000 rpm for 20 min at 4 °C, and 100 μL of supernatant was added into the detection bottle for LC-MS detection [46]. QC samples were prepared by pooling equal quantities (10 mg × 14 mice) of all test samples from both control and infected groups. QC samples were processed in the same manner as fecal samples to ensure identical treatment.

Pulmonary viral titers were determined by TCID_50_ assay. Mice were intranasally infected with IAV. At the indicated time points, lungs were collected and homogenized in 500 μL complete DMEM. Homogenates were frozen at −80 °C, subjected to three freeze–thaw cycles to release infectious virions, and clarified by centrifugation at 3000× *g* for 15 min at 4 °C. The resulting supernatants were used for viral titration. TCID_50_ assays were performed on MDCK cells. MDCK cells were seeded in 96-well plates and cultured to confluence. Lung supernatants were serially diluted in infection medium and added to the cells. Plates were incubated at 37 °C with 5% CO_2_ for 72 h, and cytopathic effect was recorded. TCID_50_ values were calculated using the Reed–Muench method and expressed as TCID_50_/mL of lung homogenate.

### 4.3. LC-MS Data Collection

Fecal metabolic profiles and all standards spectra were collected using an ultra-high-performance liquid chromatography system (Shimadzu, Tokyo, Japan) coupled to an electrospray ionization tandem mass spectrometer (AB SCIEX ZENO Q-TOF 7600, SCIEX, Singapore) with a spray voltage of 5.5 kV in positive and collision-induced dissociation (CID) mode. An increasing linear gradient of solvent A1 (0.1% formic acid in water)/A2 (0.1% formic acid in acetonitrile) (*v*/*v*) was used as follows: 0–1 min, 5% A2; 1–6 min, 5–50% A2; 6–21 min, 50–98% A2; 21–26 min, 98% A2; 26.5 min, 5% A2; and 30 min, 5% A2. The injection volume was 2 μL with a 0.3 mL/min flow rate using a C18 column (Thermo Acclaim™ 120 C18, 2.1 × 100 mm, 5 μm, 120 Å, Thermo Fisher Scientific, Waltham, MA, USA). QC samples were injected at the beginning of the sequence (three injections for column conditioning and system stabilization), after every 7 fecal sample tests, and at the end of the sequence (two injections). A total of 6 QC injections were performed throughout the analytical run. The source conditions of LC-MS were as follows: workflow, small molecule; ion source gas 1, 50 psi; ion source gas 2, 50 psi; curtain gas, 35 psi; temperature, 500 °C; TOF MS, 100–2000 Da; TOF MSMS, 20–2000 Da; collision energy, 50 V; CE spread, 25 V (stepped energy); and resolution, 42,000.

### 4.4. Raw LC-MS Data Format Conversion

The raw LC-MS data were converted to .mzXML format using MSConvertGUI software (ProteoWizard version 3.0.22050 [48,49]); the parameter settings for MS^1^ and MS^2^ data were as follows: (i) for MS^1^ data: peak picking, 1-1; subset, 1-1; threshold, absolute 100-most-intense; binary encoding precision, 32 bits; (ii) for MS^2^ data: peak picking, 2-2; subset, 2-2; threshold, absolute 20-most-intense; binary encoding precision, 32 bits.

### 4.5. LC-MS Data Preprocessing

Preprocessing for peak detection and integration, signal drift monitoring, peak and retention time alignment, removal of irrelevant background signals, adduct calculation, HMDB-matching [30,31], etc., were conducted using the FUNEL module [17] from the website NPCompass (https://npcompass.zju.edu.cn, accessed on 10 June 2026) with the following settings: ppm, 20; delta mz, 0.01; rt check, 30 s; rt tolerance, 60 s; signal intensity thresholds for controls/samples, 1000; noise level for controls/samples, 100; intensity threshold for adduct calculation, 1000; min peak width, 9; max peak width, 20; signal-to-noise thresholds, 10; compound library to search, HMDB; others, as defaults. The MS^1^ data obtained from the solvent was used as a control file for background subtraction, exclusion of irrelevant signals and feature filtering.

A .csv format file named *FilteredList* from FUNEL containing the mass-to-charge ratio, retention time, peak intensity, the result of the database match, etc., was downloaded as a feature table for subsequent data analysis.

### 4.6. LC-MS Data Analysis

MetaboAnalyst [18], which integrates data normalization, univariate analysis (*t*-test, FC), multivariate analysis (OPLS-DA, VIP), and false discovery rate (FDR) correction, was used for data analysis. Features in *FilteredList* were retained if they were present in <50% missing values (corresponding to more than 4 occurrences within 7 days), and missing values were imputed with the minimum detected value. Subsequently, Pareto scaling was applied to reduce the relative importance of large intensities while remaining sensitive to biologically relevant, low-abundance metabolites. These procedures were performed using the Normalization function in MetaboAnalyst 4.0 R with parameter settings as follows: row Norm, NULL; trans Norm, Log Norm; scale Norm, ParetoNorm.

PCA score plots of QC samples showed tight clustering, and no significant batch effect was observed. Therefore, no additional batch correction was performed. Differential abundance analysis between H1N1-infected and PBS-treated groups was performed using a *t*-test. Metabolites with VIP > 1 and |log_2_FC| > 1 were retained as initial candidates. Due to the limited sample size and the exploratory nature of this study, we did not apply multiple testing correction (e.g., FDR) at early stage, as it may have been overly conservative and increased the risk of false negatives. Instead, *p*-values (unadjusted) were used solely for ranking the candidate metabolites to prioritize those with the strongest statistical evidence for downstream analyses. To account for multiple comparisons at the final stage, *p*-values were adjusted using the Benjamini–Hochberg (BH) [50] procedure to control the false discovery rate, and q-values are shown in Table S1.

### 4.7. Structural Annotation and Validation

The MS^2^ data were annotated using SIRIUS (version 5.8.6) [18] and CANOPUS (version 5.8.6) [9], and validated by sim-Rank-Network [17], CFM-ID [23,24], and Mirror plot [21,22].

The parameters settings were as follows:

(i) For SIRIUS: instrument, Q-TOF; MS^2^ mass accuracy (ppm), 20; MS/MS isotope scorer, ignore; possible ions, all; use database formulas only, none; predict fingerprints, on; fallback adducts, all; score threshold, on; search databases, PubChem [35]; tag lipids, on; CANOPUS, on.
(ii) For sim-Rank-Network: RT tolerance for MS^2^ spectra merging, 30 s; ppm, 20; PM tolerance, 300; delta *m*/*z*, 0.01; merge spectra by collision energy, true; minimum *m*/*z* number, 5; remove precursor, true; hit number, 20; score threshold, 15.
(iii) For CFM-ID peak assignment module: spectra type, ESI; ion mode, positive; mass tolerance, 20 ppm;
(iv) For Mirror plot: peak intensity normalization; output, cosine similarity.

## Figures and Tables

**Table 1:** Workflow summary of metabolite annotation.

Step	Description	Number	Tool
1	Features after peak detection and filtering	3602	FUNEL
2	Removing signals with >50% missing values	1233	PyCharm 2024.2
3	Differential features		
3.1	VIP > 1, |log_2_FC| > 1	512	MetaboAnalyst 4.0R
3.2	VIP > 1, |log_2_FC| > 1, *p* < 0.05	40	MetaboAnalyst 4.0R
4.1	Successfully classified metabolites	38	CANOPUS 5.8.6
4.2	Successfully structure-annotated metabolites	38	SIRIUS 5.8.6
5	Metabolites annotated as lipids		
5.1	Metabolites annotated as lipids: fatty acids	7	SIRIUS, CANOPUS
5.2	Metabolites annotated as lipids: bile acids	11	SIRIUS, CANOPUS
6.1	Metabolites annotated with high confidence	1	CFM-ID, Mirror plot
6.2	Metabolites clustered with bile acid standards	7	sim-Rank-Network

## Data Availability

The data supporting this work is available in the article and Supplementary Materials.

## Supplementary Materials

The following supporting information can be downloaded at https://www.mdpi.com/article/10.3390/molecules31132275/s1. Figure S1: The principal component analysis (PCA) score plot; Figure S2: Structural annotation results of 25 downregulated potential metabolic markers from SIRIUS; Figure S3: Structural annotation results of 15 upregulated potential metabolic markers from SIRIUS; Figure S4: Low-energy-input MS^2^ spectrum (30 V) of *m*/*z* 440.2770 for CFM-ID; Figure S5: Medium-energy-input MS^2^ spectrum (55 V) of *m*/*z* 440.2770 for CFM-ID; Figure S6: High-energy-input MS^2^ spectrum (80 V) of *m*/*z* 440.2770 for CFM-ID; Figure S7: Original spectrum of *m*/*z* 440.2770; Table S1: Details of VIP, fold change, *p*-value and q-value of 40 metabolites; Table S2: Original ClassyFire superclass classification results for potential metabolic markers using CANOPUS in SIRIUS software; Table S3: Original NPClassifier superclass classification results for potential metabolic markers using CANOPUS in SIRIUS software; Table S4: Original compound annotation results for potential metabolic markers using SIRIUS; Table S5: Original peak assignment results of *m*/*z* 440.2770 using CFM-ID.
