# Supplementary material for: Structure-Based Comparative Metabolomics Identifies LysoPE 15:0 as a Candidate Metabolite Marker of Influenza Virus Infection Dynamics
Source: Molecules. 2026 Jun 29;31(13):2275. doi: 10.3390/molecules31132275 (PMC13363680; doi:10.3390/molecules31132275)
Supplement: Supplementary file 1 [file molecules-31-02275-s001.zip › molecules-4343970-supplementary.pdf]

✧ Supplementary information for

Article

Structure-Based Comparative Metabolomics Identifies LysoPE 15:0 as a Candidate Metabolite Marker of Influenza Virus Infection Dynamics

Junxiao Wang [1,†], Yuting Li [2,†], Bin Wang [3], Wenxia Fang [3], Yushen Du [2,*] and Fei Xu [1,*]

1 Zhejiang Provincial Key Laboratory of Synthetic Biotechnology for Microbial Medicine, Department of Gastroenterology, Second Affiliated Hospital, Zhejiang University, Hangzhou 310058, China; 15092094078@163.com
2 Cancer Institute, Second Affiliated Hospital, School of Medicine, Zhejiang University,
Hangzhou 310058, China; 12218164@zju.edu.cn
3 Institute of Biological Science and Technology, Guangxi Academy of Sciences, Nanning 530007, China; bwang@gxas.cn (B.W.); wfang@gxas.cn (W.F.)
* Correspondence: lilyduyushen@zju.edu.cn (Y.D.); fxu23@zju.edu.cn (F.X.)
† These authors contributed equally to this work.

Contents

Figure S1. The principal component analysis (PCA) score plot. 2

Figure S2. Structural annotation results of 25 downregulated potential metabolic markers from SIRIUS. 3

Figure S3. Structural annotation results of 15 upregulated potential metabolic markers from SIRIUS. 4

Figure S4. Low energy input MS^2 spectrum (30V) of *m*/*z* 440.2770 for CFM-ID. 4

Figure S5. Medium energy input MS^2 spectrum (55V) of *m*/*z* 440.2770 for CFM-ID. 4

Figure S6. High energy input MS^2 spectrum (80V) of *m*/*z* 440.2770 for CFM-ID. 5

Figure S7. Original spectrum of *m*/*z* 440.2770. 5

Table S1. Details of VIP, fold change, *p*-value and *q*-value of 40 metabolites. 6

Table S2. Original ClassyFire superclass classification results for potential metabolic markers using CANOPUS in SIRIUS software. 7

Table S3. Original NPClassiFier superclass classification results for potential metabolic markers using CANOPUS in SIRIUS software. 8

Table S4. Original compound annotation results for potential metabolic markers using SIRIUS. 9

Table S5. Original peak assignment results of *m*/*z* 440.2770 using CFM-ID. 10

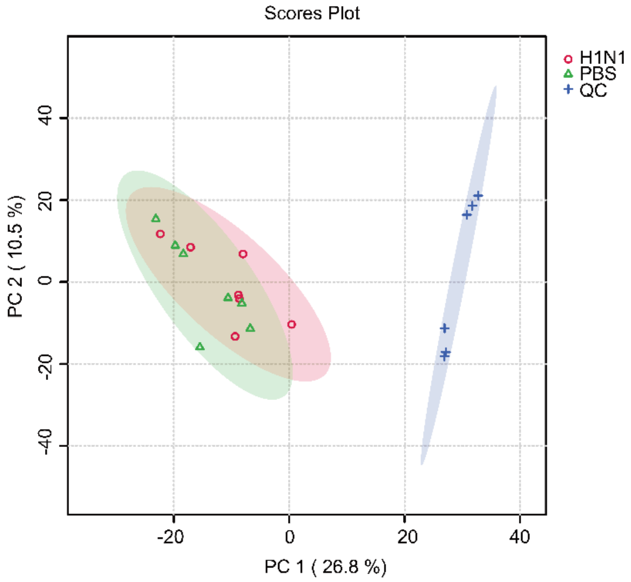

Figure S1. The principal component analysis (PCA) score plot. PCA was performed to visualize the overall metabolic differences among groups during 7 days. Each symbol represents an individual sample (one day), with different colors and shapes indicating distinct experimental groups, H1N1-infected in red, and PBS-treated in red and quality control samples in blue. The first two principal components (PC1 and PC2) are shown, with the percentage of variance explained by each component indicated in parentheses. The ellipses represent the 95% confidence intervals for each group.

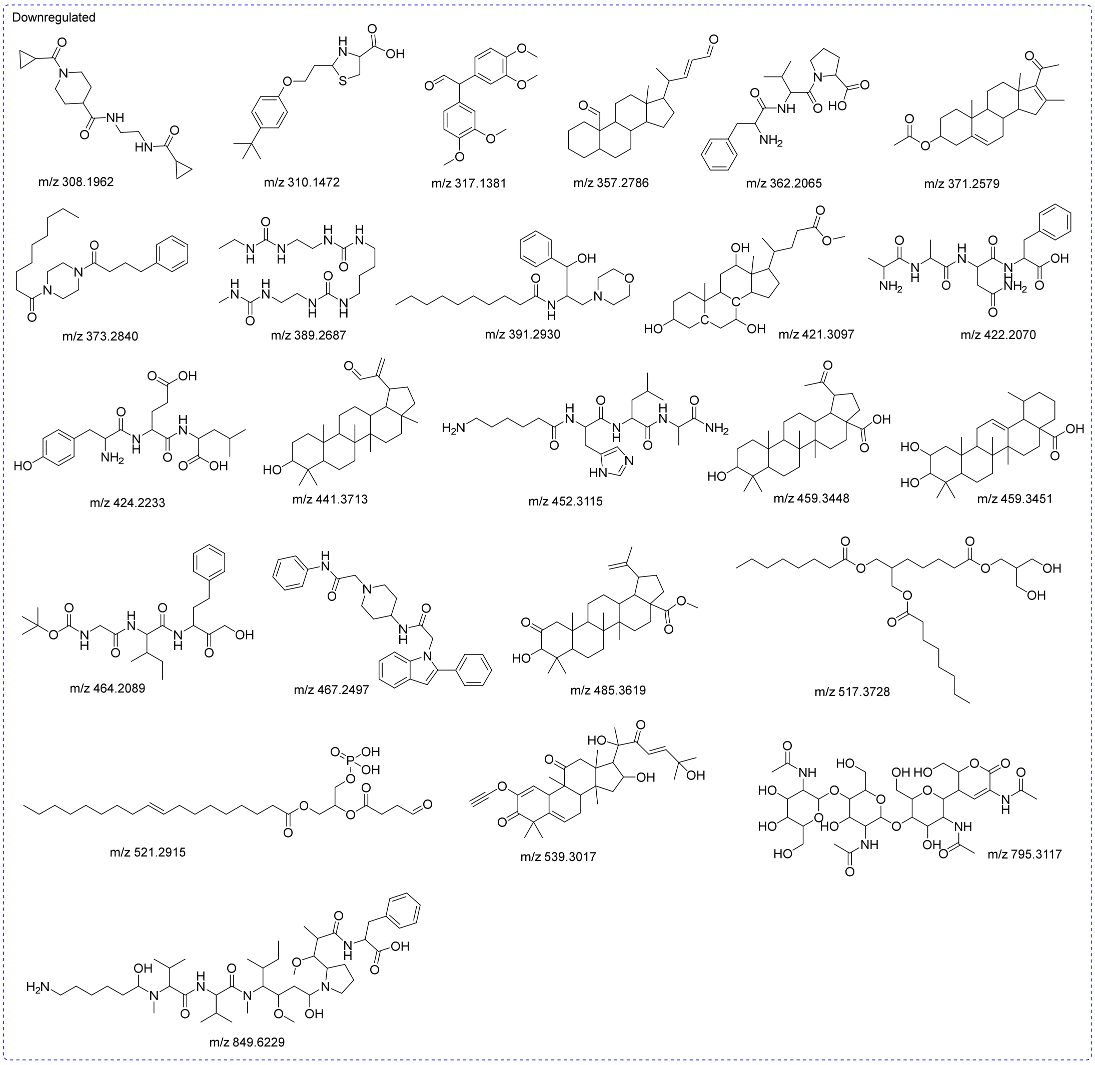

Figure S2. Structural annotation results of 25 downregulated potential metabolic markers from SIRIUS.

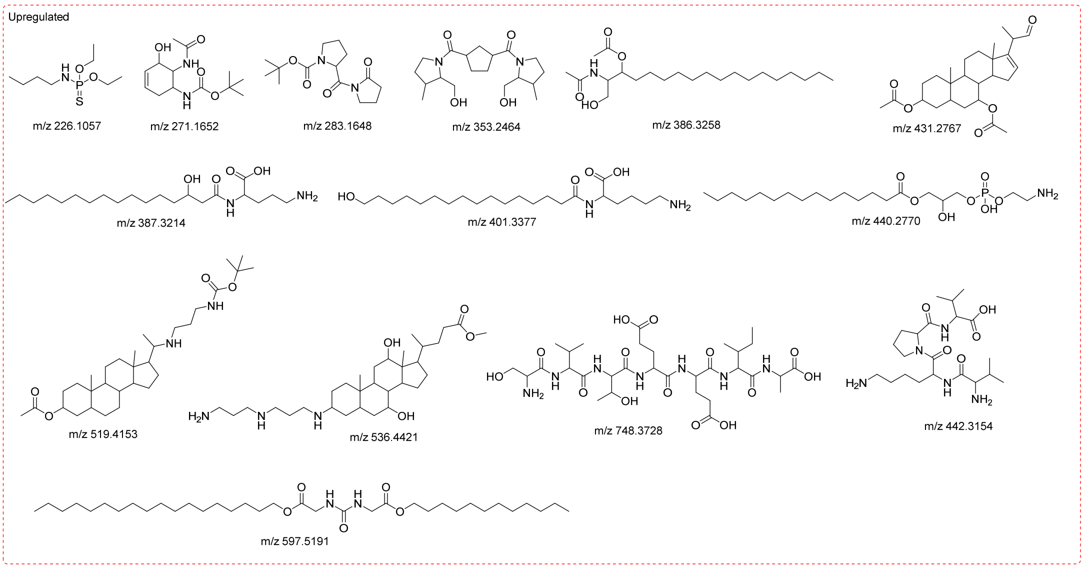

Figure S3. Structural annotation results of 15 upregulated potential metabolic markers from SIRIUS.

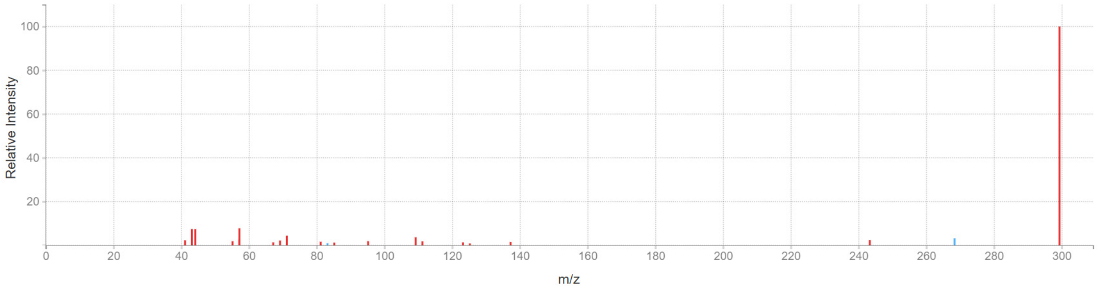

Figure S4. Low energy input MS² spectrum (30V) of *m/z* 440.2770 for CFM-ID. Input spectra are shown below. Peaks for which corresponding fragments have been found are colored red; unassigned peaks are colored blue.

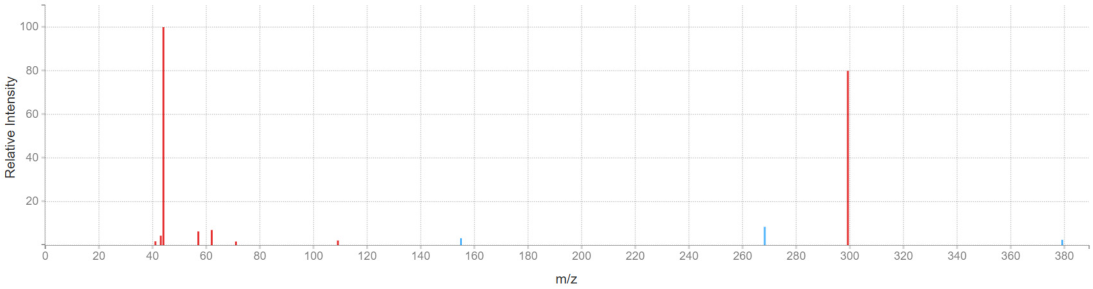

Figure S5. Medium energy input MS² spectrum (55V) of *m/z* 440.2770 for CFM-ID. Input spectra are shown below. Peaks for which corresponding fragments have been found are colored red; unassigned peaks are colored blue.

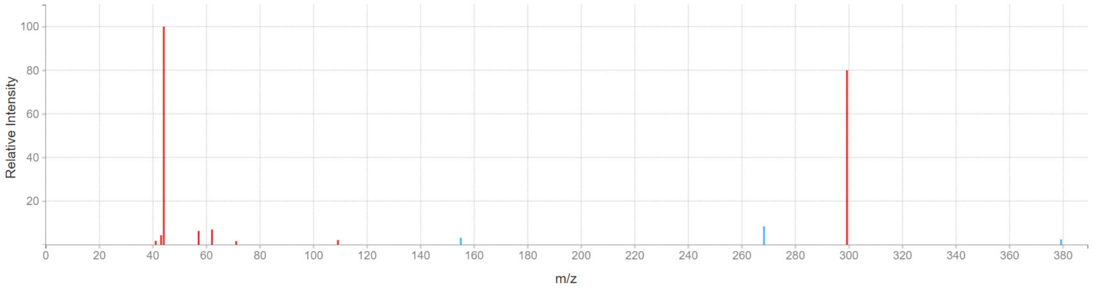

Figure S6. High energy input MS^2 spectrum (80V) of *m*/*z* 440.2770 for CFM-ID. Input spectra are shown below. Peaks for which corresponding fragments have been found are colored red; unassigned peaks are colored blue.

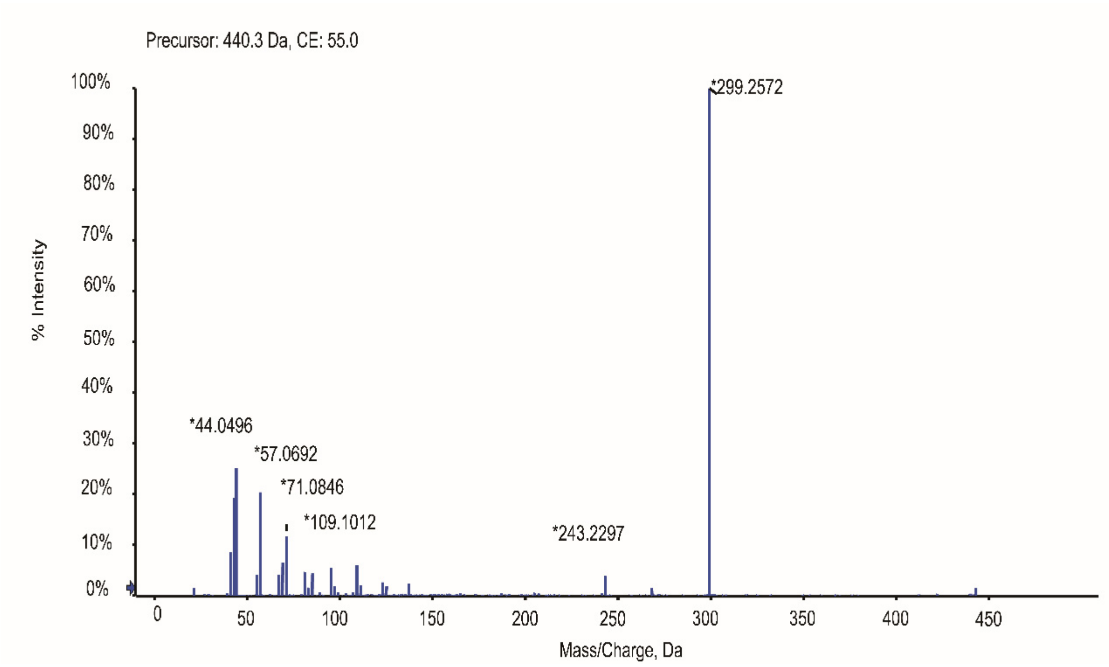

Figure S7. Original spectrum of *m*/*z* 440.2770. As METLIN is a commercially licensed database, we are unable to directly provide the raw spectrum for MID 62289. The parameter settings of two spectra were described as follows. For *m*/*z* 440.2770: instrument, SCIEX 7600 Zeno TOF; collision energy, 55V; CE spread, 25V; ion mode, positive. And for MID 62289: instrument, Agilent Q-TOF; energy, 10V, 20V, 40V; ion mode, positive.

Table S1. Details of VIP, fold change, *p*-value and *q*-value of 40 metabolites.

m/z	VIP	Log_2FC	*P* value	*Q* value	Note
226.1057	2.5572	1.4454	0.0212	0.33778	Upregulated
271.1652	1.7444	1.6662	0.0115	0.76784	Upregulated
283.1648	2.0261	1.0804	0.0284	0.76784	Upregulated
308.1962	3.2362	-1.43	3.34E-6	0.000044154	Downregulated
310.1472	2.4237	-2.024	0.0041	0.50958	Downregulated
317.1381	1.6848	-1.93	0.0430	0.76784	Downregulated
353.2464	2.3896	1.0069	0.0429	0.76784	Upregulated
357.2786	1.4751	-1.2524	0.0379	0.76784	Downregulated
362.2065	2.3116	-1.6706	0.0303	0.50958	Downregulated
371.2579	2.7332	-1.8076	0.0008	0.14161	Downregulated
373.2840	2.6248	-1.0730	0.0120	0.23002	Downregulated
386.3258	2.1094	1.0492	0.0250	0.67536	Upregulated
387.3214	2.4504	2.3526	0.0009	0.38995	Upregulated
389.2687	2.7259	-1.7509	0.0011	0.76784	Downregulated
391.2930	2.8951	-2.2945	0.0007	0.060056	Downregulated
401.3377	3.1123	2.2038	0.0001	0.0027784	Upregulated
421.3097	1.5096	-1.1778	0.0345	0.98456	Downregulated
422.2070	2.1195	-1.5811	0.0013	0.66219	Downregulated
424.2233	1.9826	-1.3756	0.0009	0.76784	Downregulated
431.2767	2.2474	2.3459	0.0381	0.568	Upregulated
440.2769	2.9813	2.0726	0.0007	0.022367	Upregulated
441.3713	1.3345	-1.0316	0.0024	0.76784	Downregulated
442.3154	2.0366	1.0159	0.0148	0.76278	Upregulated
452.3115	2.0074	-2.9071	0.0212	0.71058	Downregulated
459.3448	1.3352	-1.2806	0.0331	0.76784	Downregulated
459.3451	2.2814	-1.5340	0.0025	0.568	Downregulated
464.2809	2.5396	-2.0761	0.0015	0.34802	Downregulated
467.2497	1.4236	-1.7465	0.0468	0.76784	Downregulated
485.3619	2.6113	-1.0623	0.0039	0.22646	Downregulated
511.3029	1.5231	1.0673	0.0252	0.76784	Upregulated
517.3728	2.3874	-1.6936	0.0385	0.49528	Downregulated
519.4153	1.8084	1.2619	0.0185	0.76784	Upregulated
521.2915	1.485	-1.6819	0.0430	0.76784	Downregulated
536.4421	1.6962	1.3997	0.0326	0.76784	Upregulated
539.3017	2.2739	-1.8652	0.0352	0.59014	Downregulated
597.5191	1.896	2.6848	0.0252	0.76784	Upregulated
748.3728	2.3198	1.9395	0.0342	0.54265	Upregulated
795.3117	2.3216	-1.1215	0.0255	0.50958	Downregulated
845.4625	2.7163	-1.0803	0.0243	0.14161	Downregulated

Table S2. Original ClassyFire superclass classification results for potential metabolic markers using CANOPUS in SIRIUS software. For each metabolite, CANOPUS outputs the classification result with the highest score, detailed scores.

m/z	ClassyFire superclass	ClassyFire superclass probability	InChIkey 2D
226.1057	Organic acids and derivatives	0.961808145	KAHLOURVTYJQEO
271.1652	Organic acids and derivatives	0.977889836	WYLWNKFDZLBDJE
283.1648	Organic acids and derivatives	0.999078155	AQEKHHOQFGOZGO
308.1962	Organic acids and derivatives	0.997599423	XRKFZNHMTJTWLW
310.1472	Organic acids and derivatives	0.999795616	CPNVHBKCWFZDTL
317.1381	Benzenoids	0.999608934	VJKCVKOWXRDRJE
353.2464	Organic acids and derivatives	0.999964237	DQRPFEDLWOFKOK
357.2786	Lipids and lipid-like molecules	0.998936355	YPWICTAFCOAMFY
362.2065	Organic acids and derivatives	1.000000000	VIIRRNQMMIHYHQ
371.2579	Lipids and lipid-like molecules	0.99557966	NELMHAIFXJZDST
373.2840	Organic acids and derivatives	0.983760238	QYSUSADEYXXLAE
386.3258	Organic acids and derivatives	0.999999046	GIMYNFALDVRKIW
387.3214	Organic acids and derivatives	0.999913931	UUMBWNXCQGDFKZ
389.2687	Organic acids and derivatives	0.982474625	BAYBMBWVVFAQRM
391.2930	Organic acids and derivatives	0.881379128	UYNCFCUHRNOSCN
401.3377	Organic acids and derivatives	0.99170953	PXHONNGMOGKDCB
421.3097	Lipids and lipid-like molecules	0.914306819	WXXJILWJSSCHNC
422.2070	Organic acids and derivatives	0.999995708	HOUFWACVDOJYMT
424.2233	Organic acids and derivatives	0.999911666	NZFCWALTLNFHHC
431.2767	Lipids and lipid-like molecules	0.996625304	AGKWVTXAZOKJNC
440.2770	Organic acids and derivatives	0.999993563	FGHPDPYCRTXYNZ
441.3713	Lipids and lipid-like molecules	0.968739629	RYHMCEZDZZBPAQ
442.3154	Lipids and lipid-like molecules	0.993140996	XNKJEOHXMQNSQF
452.3115	Organic acids and derivatives	0.999990225	DPVLZJXMUGCKTN
459.3448	Lipids and lipid-like molecules	0.962662339	RVMPLOSJMIQORE
459.3451	Lipids and lipid-like molecules	0.993705094	GZCGDKJQYYABSE
464.2809	Lipids and lipid-like molecules	0.996414185	LMHGAPUYGQGRGO
467.2497	Organic acids and derivatives	0.986798346	GCBGHNTYQKTICV
485.3619	Lipids and lipid-like molecules	0.959887028	DPBNTRBGWQGODY
517.3728	Lipids and lipid-like molecules	0.964117944	OEMGYAGNRKAVNY
519.4153	Lipids and lipid-like molecules	0.991323233	SBLUYBUQVBOUAJ
521.2915	Lipids and lipid-like molecules	0.985227942	LRGUKMWMPRQYIR
536.4421	Lipids and lipid-like molecules	0.999172866	NOQLNIKEGACJSV
539.3017	Lipids and lipid-like molecules	0.970721602	VXEMRDLOFOYOJS
597.5191	Organic acids and derivatives	0.999228954	AHXAQPOBTPHTDC
748.3728	Organoheterocyclic compounds	0.888820887	LBGLUJLXGBFVQG
795.3117	Organic acids and derivatives	0.999999642	ZLYMFDKTRUDLFL
849.6228	Organic acids and derivatives	0.999945641	WWCFPVGZJJDPSM

Table S3. Original NPClassiFier superclass classification results for potential metabolic markers using CANOPUS in SIRIUS software. For each metabolite, CANOPUS outputs the classification result with the highest score, detailed scores.

m/z	NPClassiFier pathway	ClassyFire superclass probability	InChIkey 2D
146.0594	Alkaloids	0.915026903	LISFMEBWQUVKPJ
226.1057	Fatty acids	0.851990402	KAHLOURVTYJQEO
243.2100	Alkaloids	0.563023388	FUAVPNJFIZQQKC
271.1652	Amino acids and Peptides	0.430205047	WYLWNKFDZLBDJE
283.1648	Alkaloids	0.792125702	AQEKHHOQFGOZGO
308.1962	Alkaloids	0.933532178	XRKFZNHMTJTWLW
310.1472	Amino acids and Peptides	0.4680655	CPNVHBKCWFZDTL
317.1381	Shikimates and Phenylpropanoids	0.925203383	VJKCVKOWXRDRJE
353.2464	Alkaloids	0.544029236	DQRPFEDLWOFKOK
357.2786	Terpenoids	0.934449553	YPWICTAFCOAMFY
362.2065	Amino acids and Peptides	0.999032259	VIIRRNQMMIHYHQ
371.2579	Terpenoids	0.967175841	NELMHAIFXJZDST
373.2840	Alkaloids	0.884477198	QYSUSADEYXXLAE
386.3258	Fatty acids	0.691549063	GIMYNFALDVRKIW
387.3214	Fatty acids	0.871470809	UUMBWNXCQGDFKZ
389.2687	Amino acids and Peptides	0.471120894	BAYBMBWVVFAQRM
391.2930	Alkaloids	0.386546016	UYNCFCUHRNOSCN
401.3377	Fatty acids	0.696090102	PXHONNGMOGKDCB
421.3097	Terpenoids	0.977463663	WXXJILWJSSCHNC
422.2070	Amino acids and Peptides	0.998828948	HOUFWACVDOJYMT
424.2233	Amino acids and Peptides	0.954832673	NZFCWALTLNFHHC
431.2767	Terpenoids	0.996594608	AGKWVTXAZOKJNC
440.2770	Fatty acids	0.998530269	FGHPDPYCRTXYNZ
441.3713	Terpenoids	0.997796059	RYHMCEZDZZBPAQ
442.3154	Amino acids and Peptides	0.1573219	XNKJEOHXMQNSQF
452.3115	Amino acids and Peptides	0.998208761	DPVLZJXMUGCKTN
459.3448	Terpenoids	0.996624112	RVMPLOSJMIQORE
459.3451	Terpenoids	0.998289645	GZCGDKJQYYABSE
464.2809	Terpenoids	0.898769736	LMHGAPUYGQGRGO
467.2497	Alkaloids	0.985425949	GCBGHNTYQKTICV
485.3619	Terpenoids	0.998930156	DPBNTRBGWQGODY
517.3728	Polyketides	0.98733449	OEMGYAGNRKAVNY
519.4153	Alkaloids	0.871863604	SBLUYBUQVBOUAJ
521.2915	Fatty acids	0.946588993	LRGUKMWMPRQYIR
536.4421	Terpenoids	0.980130315	NOQLNIKEGACJSV
539.3017	Terpenoids	0.984818339	VXEMRDLOFOYOJS
597.5191	Fatty acids	0.947033525	AHXAQPOBTPHTDC
748.3728	Amino acids and Peptides	0.600503683	LBGLUJLXGBFVQG

795.3117	Shikimates and Phenylpropanoids	0.338414788	ZLYMFDKTRUDLFL
849.6228	Amino acids and Peptides	0.82550472	WWCFPVGZJJDPSM

Table S4. Original compound annotation results for potential metabolic markers using SIRIUS.

m/z	Confidence score	CSI:FingerID score	SIRIUS score	InChIkey 2D
226.1057	0.091738936	-60.14080422	37.17082367	KAHLOURVTYJQEO
271.1652	0.092534178	-138.5968957	77.15900389	WYLWNKFDZLBDJE
283.1648	0.303637201	-91.30813174	5.519467947	AQEKHHOQFGOZGO
308.1962	0.166450347	-139.0552976	29.23106101	XRKFZNHMTJTWLW
310.1472	0.069545856	-167.3681668	28.88504146	CPNVHBKCWFZDTL
317.1381	0.100412434	-89.35791834	71.77302042	VJKCVKOWXRDRJE
353.2464	0.078293077	-190.4663901	70.74616251	DQRPFEDLWOFKOK
357.2786	0.080160162	-82.8151203	78.32779885	YPWICTAFCOAMFY
362.2065	0.267031332	-44.90206809	29.54304794	VIIRRNQMMIHYHQ
371.2579	0.096405116	-68.18498535	115.0155771	NELMHAIFXJZDST
373.2840	0.159358932	-127.2203576	7.219807504	QYSUSADEYXXLAE
386.3258	0.299799953	-52.75212225	44.11891096	GIMYNFALDVRKIW
387.3214	0.459683124	-84.81853479	31.63182577	UUMBWNXCQGDFKZ
389.2687	0.112801323	-195.7326241	72.22430336	BAYBMBWVVFAQRM
391.2930	0.205181336	-121.5983751	42.47247111	UYNCFCUHRNOSCN
401.3377	0.103521712	-109.5037778	27.6623511	PXHONNGMOGKDCB
421.3097	0.176220759	-99.2196914	73.25379928	WXXJILWJSSCHNC
422.2070	0.09594639	-95.22756537	20.98778753	HOUFWACVDOJYMT
424.2233	0.205550104	-109.4058875	28.43611444	NZFCWALTLNFHHC
431.2767	0.102314276	-114.7158371	6.044181936	AGKWVTXAZOKJNC
440.2770	0.684791496	-1.689130822	6000.414647	FGHPDPYCRTXYNZ
441.3713	0.108696538	-56.1948941	75.19709877	RYHMCEZDZZBPAQ
442.3154	0.128400716	-119.1512869	38.91950412	XNKJEOHXMQNSQF
452.3115	0.688249764	-141.4648166	13.72211489	DPVLZJXMUGCKTN
459.3448	0.123327102	-51.17484817	143.1748996	RVMPLOSJMIQORE
459.3451	0.151891559	-45.56005387	116.682948	GZCGDKJQYYABSE
464.2809	0.047608534	-308.9498476	80.51261407	LMHGAPUYGQGRGO
467.2497	0.201228735	-182.2190451	21.57331146	GCBGHNTYQKTICV
485.3619	0.098089734	-78.27165145	147.2648719	DPBNTRBGWQGODY
517.3728	0.216290015	-94.94252832	79.68005149	OEMGYAGNRKAVNY
519.4153	0.090592949	-223.2553683	20.24064052	SBLUYBUQVBOUAJ
521.2915	0.356656992	-186.7120817	116.7336975	LRGUKMWMPRQYIR
536.4421	0.127887182	-421.603198	46.03060096	NOQLNIKEGACJSV
539.3017	0.14389972	-155.7745609	87.80171532	VXEMRDLOFOYOJS
597.5191	0.062459264	-341.344074	29.80978318	AHXAQPOBTPHTDC
748.3728	0.148231278	-379.8574708	20.49267251	LBGLUJLXGBFVQG
795.3117	0.435770205	-384.7701441	31.86746901	ZLYMFDKTRUDLFL

849.6228	0.219697683	-484.879275	29.60905031	WWCFPVGZJJDPSM

Table S5. Original peak assignment results of *m*/*z* 440.2770 using CFM-ID.

Fragment ion peak	SMILES	InChIkey
44.0495	C=C[NH3+]	UYMKPFRHYYNDTL-UHFFFAOYSA-O
57.0699	CCC=[CH3+]	FVDHQYYPYPVVLU-UHFFFAOYSA-N
62.0600	[NH3+]CCO	HZAXFHJVJLSVMW-UHFFFAOYSA-O
109.1012	[CH2+]#C/C=C/CCCC	QJHYNFBXUGBLBZ-FNORWQNLSA-N
155.1794	CCCCCCCCCC=[CH3+]	PFYPUQPBNVGOTH-UHFFFAOYSA-N
270.0737	C#CC(OCC(COP(O)(OCC[NH3+])O)O)=O	BPGIDVIZBSBSGK-UHFFFAOYSA-O
299.2581	C#CCCCCCCCCCCCCC(OCC(O)C)[OH2+]	ZPMCSNFYJKIMKF-UHFFFAOYSA-O
379.2244	C#CCCCCCCCCCCCCC(OCC(CO[P](O)([H+])O)O)O	LATZOMHHTKPYCM-UHFFFAOYSA-N
